# Parental support for free school lunches in Australian primary schools: associated factors and perceived barriers

**DOI:** 10.1017/S1368980023002240

**Published:** 2023-12

**Authors:** Gozde Aydin, Claire Margerison, Anthony Worsley, Alison Booth

**Affiliations:** 1 Institute for Physical Activity and Nutrition (IPAN), School of Exercise and Nutrition Sciences, Deakin University, Geelong, VIC 3217, Australia; 2 School of Exercise and Nutrition Sciences, Deakin University, Melbourne, Australia

**Keywords:** School lunch, Child nutrition, Health promotion, Primary school, Food services

## Abstract

**Objective:**

(1) To explore the feasibility of such programmes in Australia, this study examined parents’ views on free school lunch provision. (2) To examine the associations between parents’ demographic and personal characteristics and their support for free universal school lunches.

**Design:**

An online cross-sectional survey of parents.

**Setting:**

Australia, April 2021.

**Participants:**

Seven hundred and eighty-seven parents took the survey. They had a mean age of 40. The respondents were predominantly female (95 %) and had a university degree (72 %).

**Results:**

Fifty-three percentage of the respondents agreed that all students should have access to healthy and well-balanced, free school lunches. Parents were concerned about healthiness, catering, allergies and cost of school-provided school lunches. Ethnic background, universalism values and education levels were significantly associated with support for free school lunch provision. Non-native English-speaking parents were almost three times more likely to support free universal lunches in primary schools than their native English-speaking counterparts. Parents with higher universalism-concern values were more likely to endorse free lunches in primary school. However, the level of education was negatively associated with parents’ support for free school lunches.

**Conclusions:**

The survey results highlight the complexity of parental views on free school lunch provision. Parents’ concerns regarding lunches should be considered in developing school lunch programmes that meet the needs and preferences of diverse communities. These findings can be used to guide future primary school lunch provision initiatives.

Adequate nutrition is essential for the health and well-being of children and the establishment of healthy habits that can continue into adulthood^(1)^. However, the vast majority of primary school-age children in Australia do not consume a nutritionally sufficient diet^(2)^, putting them at risk of negative health outcomes such as excess weight gain^(3)^, poor mental health^(4)^, impaired academic performance^(5)^ and the development of non-communicable diseases later in life^(6)^. Given that children spend a significant portion of their time at school, this setting provides an ideal opportunity for promoting health. In particular, school meal programmes have potential to promote equity and improve the nutritional health of all school children through the provision of healthy meals during school hours^(7)^.

There is evidence to suggest that children’s diets do not align with national recommendations in school settings where discretionary choices are commonly consumed^(8)^ and home-packed lunches are shown to be lower in nutritional value than school-provided meals^(9)^. In addition, in a recent study, parents in Victoria (a state of Australia) identified barriers including cost and time to prepare a nutritious school lunchbox and felt that they were judged about the food they prepared^(10)^. Similarly, parents in Western Australia (another state of Australia) also reported that factors such as convenience, their child’s preferences, cost and food safety concerns hindered their ability to include healthier options in their child’s lunch box^(11)^. In another Australian study, parents voiced concern about the limited lunch time at schools and wanted teachers to eat their lunch with students^(12)^. It is apparent that current school lunch practices are far from optimal.

Given these aforementioned issues, universal school meals refer to a programme in which all students within a particular educational institution or jurisdiction are provided with meals, typically lunches, regardless of their socio-economic background or financial status. This type of initiative aims to ensure that every student has access to nutritious and balanced meals during their school day, promoting health, well-being and equal opportunities for learning. Implementation of such programmes has the potential to not only improve diet quality, food security and academic performance^(7)^ and reduce misbehaviour^(13)^ of some students but also reduce parental stress related to lunch preparation. A universal school lunch programme can improve academic performance by providing students with proper nutrition, leading to enhanced cognitive functioning, sustained energy levels and increased attendance^(7)^. Additionally, it can reduce misbehaviour by alleviating hunger-related distractions, promoting positive mood regulation and creating a more inclusive and equitable school environment^(13)^.

Free school meal programmes, which provide free lunches to all students, have been implemented in various countries around the world, with Sweden and Finland being the most well-known examples among economically developed countries. Publicly financed school meals were introduced in Finnish and Swedish contexts in the late 1940s^(14,15)^. Similarly, Brazil has a long history of implementing a school meal programme. The programme has an enormous reach, providing free nutritious meals to 43 million public school students^(16)^. Another example can be found in South Korea, where free school lunches were provided to primary school students attending government schools starting in 2011^(17)^ and the programme has since been expanded to include all children from middle and high schools, both from public and private schools^(16)^. Some countries are set to initiate similar programmes, such as Wales, where all children will get free school meals by 2024^(18)^. In the meantime, some other countries have been running pilot lunch programmes including the ‘Centrally Procured School Food Program’ in Ontario, Canada in 2017–2018^(19)^, the ‘School Meal Project’ in Norway in 2014–2015^(20)^ and New Zealand’s 2-year ‘Healthy School Lunch’ programme announced in 2019^(21)^.

In 2020, the first Australian pilot programme for providing lunch to primary school students was conducted in Tasmania^(22)^. It was conducted in three schools over 20 d, targeting 201 primary school children. Its evaluation revealed that it was highly valued by the school community. All three canteen managers expressed a desire to continue providing cooked lunches as part of the programme. The observed benefits of the programme included the promotion of social equity through the provision of food for all students, increased school attendance and engagement, a strong sense of community and the promotion of healthy eating. The programme has recently been expanded to thirty schools in 2022–2023^(23)^. However, in Australia, it is typical for primary school students to bring packed lunches from home or purchase food from school such as the canteen or lunch order service. The provision of free lunches at schools is currently a topic of public debate in Australia, with some politicians and political parties advocating for its implementation^(24)^. In order to effectively design and implement such programmes, it is important to conduct a needs assessment to understand the perspectives and preferences of all relevant stakeholders, including parents who have a primary role in providing food for their children and a vested interest in their well-being.

Therefore, the objective of the study was to explore Australian primary school parents’ views of the provision of free lunches at schools and, particularly, the barriers to it. The study also aimed to investigate the associations between parents’ demographic and personal characteristics and their views regarding free universal school lunches. It was hypothesised that parents’ demographic characteristics and personal values would both be associated with parents’ views of provision of free school lunches. The personal values selected for the survey were hedonism, universalism-nature and universalism-concern because previous research has shown that they are associated with people’s food-related practices and beliefs^(25,26)^. For example, studies have demonstrated that people with high universalism values are more likely to consume healthier foods^(25)^, support healthy eating policies^(27)^ and be interested in initiatives that promote fruit and vegetable consumption^(28)^. Conversely, those with high hedonism values tend to prioritise convenience over health concerns in their food choices^(29)^ and demonstrate a higher inclination towards purchasing convenience foods^(30)^. Based on these previous findings, we expected that parents with high universalism values would be more in favour of free healthy universal lunches at primary schools. To the best of the authors’ knowledge, this study is the largest survey conducted in Australia, exploring parents’ perspectives on the provision of free lunches.

## Methods

### Design and sampling

The study used mixed methods to investigate parents’ views of universal free school lunches. The quantitative component, in the form of an online cross-sectional survey, aimed to identify consistent patterns and demographic associations in parents’ views, while the qualitative part aimed to illuminate complex concepts that were unlikely to be captured by predetermined, closed-answer questions^(31)^. To analyse the data, we employed a descriptive theoretical framework. This approach aims to present a summary of a phenomenon as described by participants in their own words^(32)^. As such, the researchers stayed close to the data and to the surface or immediate meanings of words and experiences.

### Survey administration

Before participating in the survey, respondents were presented with a Plain Language Statement and were asked to confirm that they had read it and that they agreed to participate in the study. A pre-test of the survey was conducted by nine parents who did not participate in the final study. The pre-test allowed us to identify and address any issues with the wording or structure of the questions^(33)^. The survey was conducted online between March and April 2021. The survey was administered via the Qualtrics platform. To be eligible to participate, respondents had to be parents or primary caregivers of children attending Australian primary schools and currently living in Australia. The recruitment was done through both paid and unpaid strategies on social media platforms such as Facebook and Twitter. Respondents were offered the chance to win one of five $50 shopping vouchers for participating. Ethics permission was granted by the Deakin University Faculty of Health Human Ethics Advisory Group (HEAG-H 13-2021).

### Survey questionnaire

The whole survey included thirty-one closed-ended questions with five associated sub-questions and seven open-ended questions. Previous qualitative studies we conducted in relation to parents’ and teachers’ views of primary school food and nutrition education and environments informed the development of the survey questionnaire^(34,35)^. The present paper reports the results from one closed-ended and one open-ended follow-up question and a variety of personal values and demographic questions (below). Additional details about the survey design are available elsewhere^(36,37)^.

The closed-ended question was: *‘Well-balanced, healthy free school lunches should be provided at school to all students.’* with the response options: strongly agree, agree, neutral, disagree and strongly disagree. The follow-up open-ended question directed at those who were either neutral or did not agree was: ‘*Why do you think that well-balanced, healthy free school lunches should not be provided at school to all students?’*

#### Personal values

Nine items were selected from the fifty-seven item Schwartz Personal Values inventory (modified to be relevant to both male and female respondents) for inclusion in the survey^(38)^. These items pertained to three specific values: universalism-nature, universalism-concern and hedonism (three items per value). The respondents were asked to consider the following question: ‘*To what extent do the following statements describe you and your approach to life*?’. They were asked to rate how well the selected personal value describes their approach to life on a 5-point Likert scale (not like me at all (1), not like me (2), a little like me (3), like me (4) and very much like me (5)). Internal reliability was calculated for each personal value using the mean ratings given to the items, and the following values were obtained: 0·79 for hedonism, 0·80 for universalism-concern and 0·82 for universalism-nature. These values indicate that the items used to measure each personal value were consistent and internally reliable. The respondents’ personal value scores were then determined by computing the mean ratings for each value.

#### Demographic characteristics

Parents were asked a series of demographic questions about their gender, age, marital status, highest level of education completed, main language spoken at home and residential postcode. The residential postcodes were used to determine the level of remoteness using the Accessibility and Remoteness Index of Australia (ARIA+). The ARIA+ indices are based on the measures of road distance between populated localities and service centres^(39)^. The respondents were grouped into two categories as ‘Major cities’ and ‘Rural and remote areas’. The term ‘rural and remote’ covers all areas outside Australia’s major cities. The socio-economic status (SES) was also determined using the residential postcode, which were mapped to the Socio-Economic Indexes for Areas (SEIFA) index of advantage and disadvantage^(40)^. In this study, low SES was defined as SEIFA decile 1–3, meaning population groups with a relatively low level of advantage and a high level of disadvantage. High SES was defined as SEIFA decile 8–10, meaning population groups with a relatively high level of advantage and a low level of disadvantage.

### Data analysis

The responses to the closed-ended questions were analysed using IBM SPSS Version 27. Descriptive statistics were calculated after merging five category responses into three as agree (strongly agree and agree), disagree (strongly disagree and disagree) and neutral. Then, the response categories were dichotomised as agree (strongly agree and agree) and disagree (strongly disagree and disagree and neutral). By merging these categories, we aimed to create a more comprehensive ‘non-agree’ category. This approach simplifies the analysis, enhances clarity and enables a more insightful examination of the overall trend in responses towards the proposed school lunch programme. Using these two categories, forward logistic regression analyses were performed to identify possible predictors of parents’ views out of the demographic and personal variables. The predictors tested were gender, parental age, main language spoken at home, parental education level, SES, geolocation and parents’ universalism-concern, marital status, universalism-nature and hedonism values. Parental age was a continuous variable, gender was dichotomous (female *v*. male), main language spoken was dichotomous (native-English-speaking *v*. non-native-English-speaking, parental education level had four categories (Year 12 or less, trade/certificate/apprentice, university degree, postgraduate degree) and SES had three categories (high SES, mid SES, low SES). A two-sided Type 1 error of 0·01 was considered as a significant difference.

**Fig. 1:**
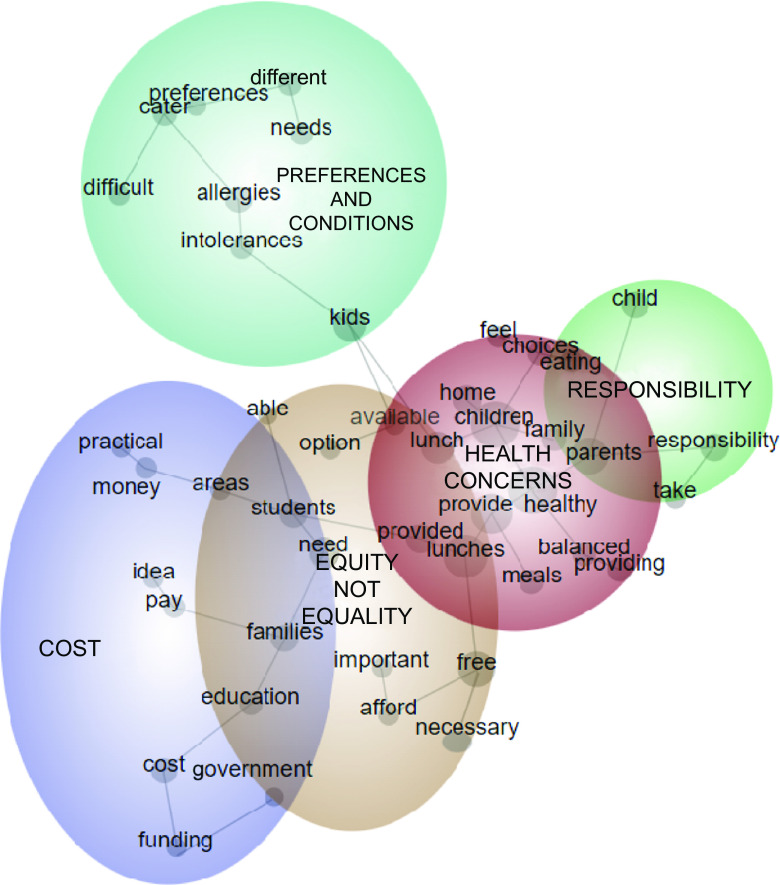
Leximancer concept map of parental views of barriers to provision of free school lunches

Open-ended responses to the questions ‘*Why do you think that well-balanced, healthy free school lunches should not be provided at school to all students*?’ were extracted from the Qualtrics database and loaded into the Leximancer software (Version 5, Leximancer Pty Ltd, 2021). Leximancer is a qualitative data analysis software that uses machine learning algorithms to automatically identify concepts and themes from text data^(41)^. Unlike manual coding, Leximancer scans textual data and automatically identifies important concepts and themes based on the frequency of word occurrences and co-occurrences^(41)^. The identified concepts are then displayed in a concept map (Fig. 1), where large circles represent the identified themes and smaller dots represent the related concepts. Leximancer labels the most prominent concepts as themes in terms of their interconnections with other concepts^(41)^. The themes are heat mapped to show their relative connectivity with other concepts in the data. Hot colours (such as red and orange) indicate the most significant themes, while (cool colours such as blue and green) symbolise less crucial themes^(42)^. The Leximancer themes were manually renamed to provide more meaningful names through the repeated reading of the respondents’ comments under each theme^(43)^. During the analysis process, GA read the parent quotations listed under Leximancer themes to create the narratives of the themes identified. Leximancer software was used along with some manual calculations to determine the exact number of mentions under each theme.

## Results

### Demographic characteristics of the respondents

Twelve hundred and fifty-nine people opened the survey link, and 787 completed the survey, resulting in a completion rate of 62 %. The respondents were predominantly female (96 %) and married (86 %), with a mean age of 40 years. Most parents had at least a university degree (72 %), and the majority spoke English as their primary language at home (93 %). The majority of the respondents were from high (54 %) or middle (37 %) socio-economic backgrounds, and most lived in major cities (66 %). The sample demonstrated reasonable representativeness compared with the Australian population, aligning with the 71 % of Australians living in major cities^(44)^. Furthermore, approximately 77 % of respondents completed their primary education in Australia, a figure broadly consistent with the 2016 Census indicating 67 % of the population being born in Australia^(45)^. However, the sample exhibited a bias towards middle and high SES and a higher proportion of respondents from Victoria (56 %). Full demographic details of the respondents were reported in a previous paper^(37)^.

### Predictors of parents’ support for free lunches in primary schools

Fifty-three percentage of parents agreed with the statement that ‘well-balanced, healthy free school lunches should be provided at school to all students’, whereas 30 % were neutral and 16 % disagreed. The stepwise logistic regression model for parents’ support for free lunches in primary schools included three statistically significant independent variables. First, non-English-speaking parents were 3·9 times as likely as English-speaking parents to support free universal lunches in primary schools (*P* = 0·0001) (Table 1). Second, for every unit increase in the universalism-concern score the odds of parents’ supporting free lunches in primary school increased by 42 % (*P* = 0·004) (Table 1). Third, the level of education was negatively associated with parents’ support for free school lunches (Table 1). Parents with trade diploma were 52 % less likely, parents with university level education were 61 % less likely and parents with postgraduate diploma were 67 % less likely to support free lunches in schools compared with parents with lowest level of education (Year 12 or less) (Table 1). Lastly, we observed that with every 1-year increase in age, the odds of parents supporting free school lunches decreased by 3 % (*P* = 0·027), although it did not reach the chosen *P*-value of 0·01 (Table 1).

**Table 1:** Associated factors of parents supporting free lunches in Australian primary schools

Variables in the equation	OR	95 % CI	*P*
Universalism-concern	1·42	1·12, 1·80	0·004*
Age	0·97	0·95, 0·99	0·027
Main language spoken	3·87	2·03, 7·39	0·000*
Education Education_year 12 or less			0·002*
Education_trade	0·48	0·26, 0·90	0·021
Education_university	0·39	0·22, 0·69	0·001*
Education_postgraduate	0·33	0·18, 0·59	0·000*

* Denotes statistical significance.

A two-sided Type 1 error of 0.01 was considered as a significant difference.

### Thematic analysis

Three hundred sixty-two parents responded to the question: ‘*Why do you think that well-balanced, healthy free school lunches should not be provided at school to all students?*’. We identified five themes that were ‘health concerns’, ‘equity not equality’, ‘responsibility’, ‘preferences and conditions’ and ‘cost’ (Fig. 1).

#### Theme 1. Health concerns

‘Health concerns’ was the most prominent theme. Many parents (*n* 47) reported that the definition of ‘healthy’ may vary between individuals, making it difficult to ensure that the food provided through a free school lunch programme would meet their expectations or the nutritional needs of their children.

*Everyone has different opinions on what is healthy. Eg. We do not have anything low fat as healthy fats are great for brain development. Parent 201*

*Some people interpret some foods to be healthy or suitable however use of ’natural’ colours or certain additives are not always true and appropriate. Parent 24*

Some parents provided examples of unhealthy food being provided at schools. One parent shared her personal experience at her child’s school as an example:

*Last year in prep, my daughter made a ’healthy lunch’ at school with a white bread roll and some veggies. Parent 109*

*Mass produced school provided lunches are historically poorer options that might not expose kids to the breadth of food options available when they see the ethnically diverse lunches that their mates are eating. Parent 354*

Many parents also expressed concern about the quality of the food that would be provided if universal lunches were implemented.

*We are very healthy eaters, I by mostly organic, preservative and additive free food, I don’t believe that the quality of food provided at school for free would match that. Parent 78*

*Most schools don’t have such healthy options in place, so I’d be worried the food wasn’t the right quality. Parent 90*

Another concern raised by parents was that the provision of free lunches at schools may discourage children and parents from learning how to prepare healthy menus and practising this skill. Many parents (*n* 43) believed that children should learn to pack their own lunches and that this practice can help to transfer food-related knowledge to the household.

*I also feel that providing healthy school lunches for free potentially could disempower parents on their journey of health. Parent 14*

*I don’t think free lunches should be provided to educate children, this does not bring the education back to the home to educate families on healthy preparation of foods. Parent 45*

#### Theme 2. Responsibility

Seventy-eight parents who were opposed to the idea of free healthy lunches at primary schools believed that it was their responsibility to provide food for their children. For example, two parents commented:

*It is not the school’s responsibility to ’parent’ a child for everything, parents have to take responsibility for some things in their children’s lives. Parent 193*

*It could be an option for those families who choose to put this responsibility in the hands of their child’s school. Parent 230*

Some parents believed that it is the responsibility of all parents to ensure the provision of healthy food for their children, regardless of their demographic characteristics:

*It is the parents’ responsibility to ensure their children are eating a healthy balanced diet. Parent 347*

*Parents of all income brackets should be accountable for feeding their child healthy food otherwise subsidising through schools won’t make a difference in the home. Parent 300*

Some parents argued that it is also the right of parents or their children to have the autonomy to make their own choices regarding food.

*Parents should make decisions about what their children eat based on their own beliefs and knowledge about well-balanced, healthy meals. Parent 56*

*Children and their parents should have the right to supply and eat their own food and make their own nutritional choices, even if they’re poor choices. Parent 76*

A smaller number of parents believed that lunch preparation was the responsibility of their children once they were capable of doing so independently.

*My children pack their own lunch and I don’t want them to lose this independence. Parent 235*

It takes away the child’s responsibility to make the right choices for their food. Parent 88

Some parents suggested alternative ways in which lunch could be provided at schools that would allow them to maintain some control over their children’s lunch options. One suggestion was to make lunches available but not mandatory for all students.

*I think it should be an option, but not mandatory. Parent 102*

*It should be accessible but not provided to all students unless reasonable to do this, and accepted by the families. Parent 291*

#### Theme 3. Equity not equality

Seventy-five parents recognised that some families may struggle to provide sufficient healthy food for their children, and they believed that schools could step in to ensure equity among students by providing free lunches for those children in need of assistance.

*I agree with providing healthy and free/subsidised lunches to school children in lower socio economic situations but as a general rule I don’t think it’s necessary. Parent 169*

*Because there are fixed resources and it is more important to focus on equity not equality. Parent 90*

Some parents believed that if lunches were provided at schools, it would not be necessary for them to be free for all students. Instead, they suggested that making lunches free for those in need would make the initiative more feasible, while allowing parents who are able to pay to do so.

*Providing lunch would be great but parents should have to pay, unless they are on a pension or experiencing financial hardship. Parent 25*

*It doesn’t need to be free, many families would be happy to pay. Parent 267*

#### Theme 4. Preferences and conditions

A number of parents (*n* 71) believed that it would be difficult and potentially unsafe to meet the needs of all children due to the variety of allergies, intolerances and sensory issues that students may have. They also cited the diversity of cultural and religious backgrounds and preferences, as well as children’s fussiness, as factors that may make it challenging to provide universal lunches that meet the needs and preferences of all students.

*It may not be feasible for so many children with allergies, intolerances and preferences to have a limited option where the school cannot cater to each individual. Parent 351*

*Very limited knowledge of allergies and sensory food issues from educators and schools not being able to adequately and safely meet these individual needs. Parent 32*

*Practically, i dont think this would work, would be hard to cater to especially in culturally diverse schools. Parent 164*

These parents believed that children with special needs may feel excluded if identical, mass-produced lunches were provided to all students.

*Very hard to cater for many food allergies and intolerances, and some kids feel left out. Parent 84*

#### Theme 5. Cost

This theme was the least prominent among the concerns raised by parents but still many parents believed (*n* 69) that subsidising school lunches would be a financial burden on taxpayers, and some were unwilling to pay the additional cost.

*I can understand there may be a need for other children, but it’s not something my children need, and there would obviously be a cost associated presumably at the taxpayers’ expense. Parent 14*

While acknowledging that some children may not have access to sufficient healthy lunches on a daily basis, some parents believed that providing these lunches to every child would not be cost-effective due to the high risk of waste and unacceptance.

*Children are very complex relative to what they do and don’t like and you can’t please them all so I think free lunches would lead to too much food wastage. Parent 90*

*While it would benefit certain children, it would be a huge burden on already stretched federal/school budgets especially when a lot of parents are able to provide it for their children. Parent 78*

Some parents argued that schools are already underfunded and have limited resources. They believed that providing universal lunches at no cost to parents could potentially take funding away from other areas such as special classes or other school expenses.

*If mandated then which areas will suffer from budget cuts or reallocation of school funds. Parent 37*

A few parents also believed that a plan for universal lunches at schools could suffer from insufficient funding, which could affect the quality of the lunches provided, based on their own experiences.

*History has taught us provided lunches at kinder may start with best intentions however have been influenced with budgets. Parent 369*

## Discussion

Overall, slightly more than half of the parents supported the provision of free school lunches, while 30 % were unsure. Non-English-speaking parents were more likely to support free universal lunches in primary schools. In addition, the higher the universalism-concern value scores of parents, the more likely they were to support free lunches in primary school. On the other hand, a higher level of education was negatively associated with support for free school lunches. Some of the concerns expressed by parents regarding the provision of free school lunches included the healthiness of the meals, loss of control over their children’s food choices, catering for children’s preferences and special dietary needs and cost.

### Parental support for free lunches

Fifty-three percentage agreed that all students should be provided with healthy, well-balanced free school lunches. However, this level of support is lower compared with another recent Australian pilot study of seventy-one parents which found 86 % of parents supported school lunch provision^(46)^. The participants in the Manson *et al.*’s study^(46)^ were predominantly highly educated females with a mean age of 40 and data on spoken language at home were not collected in this study (*personal communication*). As the characteristics of the sample in the current study generally resemble those of Manson *et al*.’s study, the observed discrepancy in support for free school lunches could potentially be attributed to differences in how the question was framed and the inclusion of the term ‘free’ in the present study. Parents in our study raised concerns about potential waste and the associated increase in taxes if school lunches were provided for free to all students. Additionally, the sample of the current study consisted primarily of native English speakers, which were less in favour of free school lunches compared with their less-educated and non-English-speaking counterparts. This could also have contributed to the lower level of overall support observed. On the other hand, upon completion of a free school lunch trial in Tasmania, 90 % of parents expressed a desire for their school to provide cooked lunches every day. This suggests that setting a good example may serve to alter the perspectives of parents who are sceptical about free lunch provision at schools.

### Predictors of parental support for free lunches

The use of a language other than English at home was found to be the most significant predictor of support for the implementation of universal free lunches at schools in Australia. This may be due to a lack of familiarity with the school-provided meal concept among parents who were born and educated in Australia, where the cultural norm has been for parents to provide food for their children through the use of lunchboxes. In contrast, school meal programmes are widespread globally, with an estimated 330 million children in 139 countries participating in such programmes in 2020^(47)^. Many of these countries also offer school meal subsidies^(47)^. It is possible that parents who have had the opportunity to observe school meal programmes in their home countries may have a greater appreciation for the practice and be more supportive of its implementation in Australia. Additionally, non-Australian parents may face more difficulties in providing healthy meals for their children, struggling to have access to guidance and advice, being unfamiliar with the school and health systems and may thus support the programme. More research is warranted to investigate this relationship further.

The universalism concern value scores of parents were positively associated with their support for universal free lunches at schools. This is expected, as the universalism value is related to a concern for the welfare of others in the larger society and world, as well as issues of social justice and equality^(48)^. Previous research has also demonstrated an association between people’s universalism values and their support for initiatives that promote fruit and vegetable consumption^(28)^ and their support for healthy eating policies^(27)^. This highlights the importance of considering value orientations and demographic factors in understanding support for social policies such as school meal programmes.

Parents with higher levels of education and older parents were less likely to support the implementation of universal free school lunches. This may be due to their higher levels of food security and their greater self-efficacy in terms of packing a healthy lunch, which could be influenced by their knowledge and experience with food. Previous studies have suggested that education is protective against food insecurity^(49)^, whereas young populations are at a higher risk of food insecurity^(50)^. It has also shown that nutritional knowledge increases with age^(51)^ and increasing levels of formal education^(51)^. Therefore, these parents may be more resistant to the idea of the government providing a service that they feel they are capable of providing themselves. Further research is needed to fully understand the motivations behind the attitudes of parents with different levels of education and of different ages towards school meal programmes.

### Parental views of barriers to implementation

The main concerns of parents were centred around the healthiness of the lunches to be provided, the loss of control over their children’s food choices, the catering for children’s preferences and special dietary and the cost.

The healthiness of the free school lunches was the primary concern among parents. To address this concern, one potential solution could be to adopt the practice of employing nutritionists to plan menus for free school meals, as is done in countries such as Brazil and Korea^(17,52)^. This would ensure that children have access to nutritious and sufficient meals that align with the Australian Guide to Healthy Eating. Additionally, parents highlighted the challenges of catering to the diverse needs of students, including food allergies, intolerances and cultural diversity, which could also be managed by nutrition professionals designing the menu. Another concern cited by parents was the potential burden on taxpayers. Previous research has also found that parents have concerns about their children getting enough to eat at lunchtime, about enjoying the food served^(53,54)^ and about the financial impact on schools^(13)^.

To address the concerns of parents and opponents, it may be useful to implement strategies such as increased transparency and communication about the menu planning and food procurement processes, as well as involving parents and other stakeholders in these decision-making processes. A universal meal subsidy programme, where the cost of providing meals to students is partially or fully covered, can be considered as an option to alleviate parental concerns regarding the potential rise in taxes. However, a cashless system or any measure that ensures confidentially could be used to minimise the associated risk of stigma^(55)^. Additionally, efforts could be made to educate parents and the wider community about the many potential benefits of school meal programmes in order to increase support for these initiatives.

Some parents felt that school lunch provision would negate the responsibility of parents and students to make healthy food choices and disempower them in their health journey. However, on the contrary, school meals can empower children providing teaching occasions in which children learn about food, meals and healthy eating, which is referred to as ‘pedagogic meals’^(56)^. In the current study, some parents suggested providing lunches only for the children in need. Although this idea can be a public discussion topic to find the most feasible way of school lunch provision, it is important to consider the potential stigma that may be associated with providing lunches only to students in need^(7)^. Drawing from a previous UK study^(55)^ that revealed the lack of stigma is linked to homogeneity and normalisation of free school meal entitlement, a feasible option could involve initiating a whole school programme in low socio-economic regions. Implementing such a programme across the entire school, rather than singling out specific students, could be a better approach to ensure inclusivity and reduce potential stigmatisation associated with free meal provision.

### Strengths and weaknesses

The main strength of this study was its focus on the views of parents from various demographic backgrounds across Australia concerning universal free school lunches. This allows for a more diverse and representative sample of views, which can provide a more comprehensive understanding of the issue. The exploration of associations between parents’ views of universal free school lunches and their demographic characteristics and personal values is also novel and valuable. By exploring these associations, the study adds to our understanding of the factors that influence parents’ views on universal free school lunches and may help to inform policy and practice related to school lunch programmes.

The main limitation of this study is related to the predominance of highly educated people in the sample. While this reflects the views of better-off parents, the views of less well-off parents may be underemphasised in the results. A broader, more demographically representative sample may hold a different mix of views about free lunch programmes.

The implications of the results for Australia are essential for policymakers and stakeholders. Understanding the factors influencing parental support for free school lunches can aid in designing effective programmes and policies to promote healthier eating habits among students. Addressing concerns raised by parents, such as healthiness and cost, through evidence-based strategies may improve the acceptance and implementation of such initiatives. Additionally, recognising the role of cultural norms and value orientations in shaping attitudes towards school meal programmes can inform communication and education efforts to garner wider support from the community. Future research should aim to broaden the representation of other demographic groups to improve the generalisability of findings and further understand the complexities surrounding parental attitudes towards free school lunches.

## Conclusion

It is important to consider the perspectives of parents in any effort to introduce free school lunches in Australian primary schools, as their support is critical to the success of such initiatives. While a majority of the parents surveyed indicated support for free lunches at primary schools, the concerns of the opposers should be taken into account. An exploration of parents' views can help inform the design and implementation of school meal programs and provide guidance for future efforts to improve the nutritional status of children.
